# Clinical evidence for allergy in orofacial granulomatosis and inflammatory bowel disease

**DOI:** 10.1186/2045-7022-3-26

**Published:** 2013-08-15

**Authors:** Pritash Patel, Jonathan Brostoff, Helen Campbell, Rishi M Goel, Kirstin Taylor, Shuvra Ray, Miranda Lomer, Michael Escudier, Stephen Challacombe, Jo Spencer, Jeremy Sanderson

**Affiliations:** 1 Departments of Gastroenterology at Guy’s & St. Thomas NHS Foundation Trust, London, UK; 2 Peter Gorer Department of Immunobiology, King’s College London School of Medicine at Guy’s Hospital, London, UK; 3 King’s College London Dental Institute, London, UK; 4 Nutritional Sciences Research, Waterloo Campus, Kings College, London, UK; 5 Department of Gastroenterology, 1st Floor, College House, St. Thomas’ Hospital, Westminster Bridge Road, London SE1 7EH, UK

**Keywords:** Allergy, Food allergens, Orofacial granulomatosis, OFG, Oral allergy syndrome, OAS, Crohn’s disease, Ulcerative colitis, Inflammatory bowel disease, IBD

## Abstract

**Background:**

Orofacial granulomatosis (OFG) causes chronic, disfiguring, granulomatous inflammation of the lips and oral mucosa. A proportion of cases have co-existing intestinal Crohn’s disease (CD). The pathogenesis is unknown but has recently been linked to dietary sensitivity. Although allergy has been suggested as an aetiological factor in OFG there are few published data to support this link. In this study, we sought clinical evidence of allergy in a series of patients with OFG and compared this to a series of patients with inflammatory bowel disease (IBD) without oral involvement and to population control estimates.

**Methods:**

Prevalence rates of allergy and oral allergy syndrome (OAS) were determined in 88 patients with OFG using questionnaires, skin prick tests, total and specific serum IgE levels. Allergy was also determined in 117 patients with IBD without evidence of oral involvement (79 with CD and 38 with ulcerative colitis (UC)).

**Results:**

Prevalence rates of allergy in patients with OFG were significantly greater than general population estimates (82% versus 22% respectively p = <0.0005). Rates of allergy were also greater in those with CD (39%) and, interestingly, highest in those with OFG and concurrent CD (87%). Conversely, whist OAS was common in allergic OFG patients (35%) rates of OAS were significantly less in patients with concomitant CD (10% vs 44% with and without CD respectively p = 0.006). Amongst CD patients, allergy was associated with perianal disease (p = 0.042) but not with ileal, ileocolonic or colonic disease location. Allergy in UC (18%) was comparable to population estimates.

**Conclusion:**

We provide compelling clinical evidence for the association of allergy with OFG whether occurring alone or in association with CD. The presence of gut CD increases this association but, conversely, reduces the expression of OAS in those with atopy. Interestingly, there is no evidence of increased allergy in UC.

## Introduction

Orofacial granulomatosis (OFG) is a chronic inflammatory disorder presenting characteristically with lip swelling. It can also affect other sites in the oral cavity such as the gingivae, buccal mucosa and the floor of the mouth [1]. Most cases of OFG present as a separate clinical entity, but a proportion present in association with systemic conditions such as intestinal Crohn’s disease (CD), sarcoidosis or the Melkersson-Rosenthal syndrome (MRS) [2-4]. Although CD and OFG share a number of clinical and histological features, the exact relationship between the two conditions is unknown [5]. Non-specific oral lesions, particularly aphthous stomatitis, are common in CD, usually reflecting periods of nutritional deficit or active disease [6,7]. Conversely, OFG-like changes, usually lip swelling or buccal involvement, are uncommon in established gut CD [8].

Clinical allergy in the form of hay fever, atopic eczema or asthma has been reported as being more prevalent, affecting 12% to 60% of OFG patients compared to 15% of the general population [9-11]. In an early study of 75 patients with OFG, the incidence of allergy was reported as 60% [12]. This compares with a recent worldwide survey (World Allergy Organization) in which the rate of allergy was reported as 22% [13]. Many diseases affecting the orofacial region have an allergic basis and the role of dietary antigens in particular in the aetiopathogenesis of OFG is compelling [14,15]. Cinnamon and benzoates have been found to be positive on patch testing in patients with a variety of oral mucosal diseases including OFG [16]. Specific dietary exclusion of cinnamon and benzoates in patients with OFG results in objective improvement in disease activity [17]. How these substances contribute to the development of OFG is as yet unclear.

Furthermore, evidence of a role for immunoglobulin E (IgE) in OFG comes from the identification of a novel subset of B cells in the oral mucosa in patients with OFG (subepithelial dendritic B cells), a proportion of which are class-switched to IgE. This may provide the link between dietary allergens and the induction of local oral inflammation [18].

OFG has clinical similarities with the Oral Allergy Syndrome (OAS), a well recognised condition with a clear immunological pathogenesis [19,20]. Pathologically, OAS is distinct in that permanent granulomatous inflammation is not a feature. OAS defines a complex of symptoms induced by exposure of the oropharyngeal mucosa to food allergens in patients who are allergic to tree pollens, in particular to silver birch. The relevant foods contain similar determinants to aeroallergens to which the susceptible individual has been previously sensitised. IgE-mediated immediate hypersensitivity reactions are clearly defined and some patients also experience delayed (>24 hrs) responses which correlate with T cell recognition [21]. These delayed reactions which follow IgE-mediated responses can last up to one week but are not associated with a granulomatous lesion.

Avoidance of the dietary triggers in OAS is the main treatment modality and largely alleviates symptoms. There are therefore some similarities between OAS and OFG in terms of symptoms, triggers and treatment regimens.

The contribution of allergy, if any, to the development of inflammatory bowel disease (IBD) has long been a matter for debate. Over 40 years ago, a greater prevalence of allergic disorders in patients with CD and ulcerative colitis (UC) compared to matched controls was demonstrated [22]. Other studies have confirmed raised levels of serum IgE in patients with IBD [23,24]. Furthermore, there has also been a long established link between lung disease and IBD [25]. Recently, an increase in allergic symptoms, respiratory symptoms, abnormal lung function and skin prick test (SPT) positivity to common allergens in patients with IBD was confirmed [26].

Other studies have been less positive regarding the association of atopy and IBD. Previous studies were unable to show any difference in atopy, as assessed by SPTs to a panel of common allergens, between patients with IBD and healthy controls [27,28]. They did however show a highly significant increase in the number of positive SPTs to food allergens. This increased response to food allergens was replicated using specific serum IgE tests in 100 patients with IBD compared to 100 matched healthy controls [29].

The aim of this study was to determine the rate of allergy in a large cohort of patients with OFG and compare this to a group of patients with IBD with no evidence of oral involvement and to reported population estimates of allergy.

## Methods

### Clinical profile

Eighty-eight consecutive patients with biopsy-proven OFG (mean age 37.1 years [range 8-84]; 43 female) were recruited from those attending a multi-disciplinary oral medicine/ gastroenterology out-patient clinic at Guy’s & St. Thomas’ NHS Foundation Trust Hospital (GSTFT, London, UK) between August 2007 and July 2009. The diagnosis of OFG was based on characteristic clinical and histological findings. Co-existent intestinal CD was diagnosed by standard endoscopic, radiological and histological criteria. In addition, a group of 117 consecutive patients with confirmed IBD but no oral involvement (mean age 39 years [range17-77 years]; 66 female) were recruited from those attending the IBD service at GSTFT (79 CD, 38 UC). Allergy testing was undertaken as part of routine clinical practice in the assessment of OFG and part of an OFG research program approved by the Local Research Ethics Committee (Approval No. 95/5/6; Lewisham & North Southwark REC).

### Allergy assessment

All patients completed an allergy questionnaire as part of their initial assessment. This included identifying asthma, eczema and allergic rhinitis as well as reactions to common allergens. SPTs to a panel of common allergens (grass, silver birch, plane, house dust mite, cat and candida) and to cinnamon and benzoate were performed. Allergen extracts from Stallergenes France were used. Histamine and saline were used as positive and negative controls respectively. Wheal size was assessed at fifteen minutes and a reaction of more than 3 mm was taken to be positive. Blood was also taken for measurement of total and specific serum IgE levels (ImmunoCAP) to 6 allergens (mixed grasses, silver birch, cat dander, house dust mite, apple and hazel nut). Specific IgE levels equal to or greater than 0.70 KU/L (Grade II) were considered positive. Clinical evidence for allergy was based on a positive history of asthma, eczema, allergic rhinitis or acute food allergy. Allergy rates were compared to population estimates [13].

### Statistical analysis

Data were analysed in the GraphPad Prism statistical PC program (GraphPad Software, San Diego, California) using the χ [2] test. A level of p < 0.05 was considered statistically significant.

## Results

### Clinical evidence for allergy

There was no significant difference in the demographics of the different patient groups studied. A positive history of allergy was observed in 81.8% (72 of 88) of patients with OFG which is significantly greater than the 22% [13] estimated for the general population (p < 0.0005). It was also significantly greater than the prevalence of allergy in patients with CD (31 of 79 patients [39%]) without oral involvement (82% vs.40.2%; p < 0.0005). The presence of OFG with concurrent CD was associated with allergy in 20 of 23 (87%) of cases, slightly higher than the rate of allergy in OFG alone and significantly greater than in patients with CD without oral involvement (87% vs 39%, p < 0.0005). Younger patients (disease onset ≤30 years of age) with OFG alone (no intestinal CD) had the highest rates of allergy (35 of 37 patients; 95%) than those presenting later in life (17 of 28 patients; 61%) (p = 0.0007), though the older patients still had a significantly higher rate of allergy than the general population. There was no difference in allergy rates amongst patients with OFG and concurrent CD when stratified according to age of disease onset.

Table 1 shows the prevalence of allergy in CD according to anatomical disease location (Montreal classification [30]) and by treatments received. Interestingly, perianal involvement, previously shown to be more frequent in patients with OFG [31] was the only location for which allergy was more frequent (p = 0.042). There was no association between allergy and need for surgery or treatment with immunosuppression or biologics (indirect markers of a more severe disease course).

**Table 1:** Prevalence of allergy in Crohn’s disease according to anatomical disease location and treatments received

**Pattern / treatment**	**Non-allergic (n = 49)**	**Allergic (n = 33)**	**P value (Χ**^**2**^**)**
Ileal only	7	3	0.72
Colon only	12	6	0.84
Ileo-colonic	31	23	0.72
Rectal	2	5	0.18
Perianal	6	11	0.04
Immunosuppression	32	24	0.64
Biologic ever	15	8	0.71
Surgery ever	30	18	0.71
> 1 resection	10	9	0.65

Conversely, only 7 of 38 (18%) of patients with UC gave a history, not significantly different to population estimates (22%) but significantly lower than that seen in patients with CD (18% vs 40.2% respectively, p = 0.024). Rates of allergy did not vary in UC according to extent of involvement in UC.

### Rates of oral allergy syndrome

Patients with OFG frequently reported immediate hypersensitivity reactions in their mouth to certain foods. The most common foods were fruits, particularly those sharing the common Bet v1 epitope responsible for OAS [19,20]. These included apple, orange, tomato, kiwi fruit, peach, melon and hazelnuts. 25 of the 72 allergic patients with OFG had evidence of OAS (35%). When the patients were separated according to the presence or absence of CD, there was significantly more OAS amongst allergic OFG patients without CD compared to those with concomitant CD (23 of 52 [44%] vs 2 of 20 [10%]; p = 0.006). The prevalence of OAS in the different patient groups is outlined in Table 2. The rate of OAS in patients with SPT or specific IgE positivity to silver birch was 43% (21 of 49 patients). This is not significantly different from previous observations in the literature [32]. The rate of OAS in those OFG patients without CD sensitised to silver birch was 49% compared to 20% in those OFG patients with concomitant CD. The presence of CD per se was therefore associated with a lower rate of OAS in all groups.

**Table 2:** Prevalence of OAS in allergic OFG patients and silver birch sensitised patients according to presence or absence of concomitant CD

**Patient group**	**OAS (%) in allergic group**	**OAS (%) in silver birch sensitised group**
**OFG alone**	23/52 (44%)	19/39 (49%)
**OFG + CD**	2/20 (10%)	2/10 (20%)
**Total**	25/ 72 (35%)	21/49 (43%)

### Clinical measures of allergy

Table 3 shows markers of allergy (SPT and serum IgE levels) in our study. As expected, mean total IgE levels were significantly raised in atopic patients with OFG whether or not they had concurrent CD (see Table 3). Atopic patients with OFG and concurrent CD had higher mean IgE levels than those with OFG alone, though this was not statistically significant (360 KU/l vs 224 KU/l p = NS). The prevalence of at least one positive SPT in allergic OFG patients (with or without CD) was 90% compared to 8% in those without allergic symptoms. Positive SPTs to grasses and tree pollen (silver birch) were the most prevalent in our patient population, present in 75% of allergic OFG patients (with or without CD). 19% and 35% of the allergic OFG patients (with and without CD respectively) were SPT positive to cinnamon. Likewise, 13% and 30% (allergic OFG patients with and without CD respectively) were SPT positive to benzoates. The prevalence of 1 or more positive SPT was 91% in allergic CD patients with no oral involvement. As expected, in the absence of reported allergy, SPT positivity was low in all groups.

**Table 3:** IgE sensitisation and skin prick tests in different patient groups

	**Allergic**	**Non-allergic**	**Allergic**	**Non-allergic**	**Allergic**	**Non-allergic**
	**OFG**	**OFG**	**OFG + CD**	**OFG + CD**	**Crohn’s alone**	**Crohn’s alone**
	**n = 52**	**n = 13**	**n =20**	**n = 3**	**n = 32**	**n = 49**
**Positive SPT result (%)**						
Grass	75	0	75	0	69	8
Silver	75	0	50	0	59	2
Birch						
Cat	44	0	60	0	59	6
Derm	54	0	65	0	72	12
Plane	52	0	35	0	47	0
Candida	33	0	60	0	44	2
Cinnamon	19	0	35	0	16	0
Benzoate	13	8	30	0	13	0
**Positive ImmunoCAP result; >Grade II**						
Grass	67	8	65	0		0
Silver	32	0	29	0		0
Birch						
Cat	34	0	35			
Derm	34	0	53	0		
Apple	6	0	18	0		
Hazel	30	0	29	0		
Mean total IgE in KU/L	224	38	360	12		
≥1 SPT positive	90	8	90	0	91	20
≥1 RAST positive	76	0	82	0		
≥ SPT +/- RAST positive	90	13	90	0		

*SPT* (Skin Prick Test), Total and Specific Serum IgE.

Positive specific serum IgE titres also correlated well with reported allergy. The prevalence of a positive test (equal to or greater than 0.70 KU/L) was 76% in allergic patients with OFG alone and 82% in allergic patients with OFG and CD. In keeping with SPT results, mixed grasses were the most prevalent of the six allergens tested. The prevalence of at least one positive SPT or at least one positive specific serum IgE was 90% in allergic OFG patients (irrespective of presence of CD) and, naturally, very low (13%) in in patients with non-allergic OFG and zero in patients with non-allergic OFG with CD.

## Discussion

The findings in this study confirm the remarkable association of allergy with OFG regardless of the presence or absence of concurrent intestinal CD. Rates of atopy have been studied in a variety of other chronic inflammatory disorders (granulomatous or not) and have not been found to be raised, including sarcoidosis [33]. Indeed, in certain conditions, for example, rheumatoid arthritis and multiple sclerosis, atopy rates are reported to be lower than the population average [34,35]. This fits with the finding in patients with UC in this study where allergy rates were no different to those reported in the general population.

The high atopy rates in OFG and CD therefore appear quite unique. In OFG, where atopy is near universal, the obvious question is whether allergy is involved in the pathogenesis of this rare condition. A role for allergy would be supported by the clinical presentation (patients frequently report swelling after specific dietary or other environmental exposure), the response to exclusion diets (e.g. cinnamon- and benzoate-free) and the novel finding on oral mucosal immunohistochemistry of a dendritic B cell infiltrate expressing IgE [26].

In gut CD without associated OFG, atopy rates are less marked but still clearly higher than population controls. An interesting question is whether the presence of atopy might in some way modify the phenotype of CD, particularly by altering the Th1/Th2 polarisation in the immune response in the gut. In this study, colonic involvement was more frequent in atopic CD compared to an ileal disease site, counter-intuitive to the fact that patients with UC, a pure colonic disease, have no increased atopy. Perianal involvement was also more frequent in atopic CD, an observation probably explained by the previous finding that OFG is, itself, associated with perianal CD more frequently than expected [31]. Immunologically, CD is conventionally considered a Th1 disease. The only study to have addressed the cell infiltrate in OFG supports a Th1 profile in this condition as well. Conversely, allergic disorders display a Th2 polarisation. In other settings, there is evidence that concurrent atopy, for example allergic asthma, modifies a co-existing chronic inflammatory disease. Likewise, helminthic infection in the gut provokes a Th2 polarised response which, intriguingly, may modify the activity of CD through a shift away from Th1 polarisation, used to advantage for example, in the administration of Trichuris suis as therapy for IBD [36].

An explanation for a link between allergy and CD may come from the hygiene hypothesis in which individuals raised in a sanitary environment are more prone to develop certain diseases in later life. It is thought that individuals who are exposed to enteric pathogens from an early age are more protected from developing CD in the future. Exposure to pathogens induces a Th1 response in early life which renders individuals less susceptible to developing Th2 disease later in life. Hence, another possibility linking OFG/CD and allergy is a shared increase in susceptibility arising from similar environmental factors early in life, including helminthic infections, antibiotic use, breastfeeding, family size/sibship, urban upbringing, personal and domestic hygiene [37-40]. This may particularly apply to the link between CD and allergy but the rates of allergy in OFG are too high to be explained simply by shared environmental influence.

In support of the hygiene hypothesis, two recent prospective studies have reported a slightly increased risk of IBD in children who receive antibiotics in their first year of life [38,39]. Further support comes from a Spanish case-control study of 270 IBD patients which concluded that better childhood living conditions are associated with an increased IBD risk [40]. Interestingly, a recent American study of 83 IBD patients and 54 healthy controls found an increased frequency of brushing, dental floss and breath freshener use at onset of IBD [41].

The rates of OAS in patients with OFG without CD are as expected from other cohort studies [32], but are much lower when OFG patients have concomitant CD. The presence of CD seems to significantly affect the clinical expression of OAS. Only 10% of allergic OFG patients with concomitant CD have OAS, compared to 44% of allergic patients with OFG alone. Only 20% of silver birch sensitised OFG patients with concomitant CD have OAS, compared to 49% of silver birch sensitised patients with OFG alone. Nevertheless, the overlap between OFG and OAS raises the intriguing possibility of both immediate and delayed hypersensitivity occurring sequentially in the same patients. This could also be a factor in the pathology of OFG.

Another feature of our cohort is that the prevalence of allergy in the CD patients is elevated even further by the presence of OFG. Indeed, in patients with OFG without CD and with a young age of disease onset, allergy is present in almost all patients (95%, 35 of 37 patients in this study). SPT positivity in non-atopic patients with OFG was comparable to that noted by Droste et al [42]. The prevalence of at least one positive SPT in allergic OFG patients (90%) was far greater than the 55.4% reported by Droste et al [42] in their most allergic group of patients. The high prevalence of at least one positive SPT or at least one positive specific serum IgE in allergic OFG patients (90%) compares with 63.1% in the most allergic group reported by Droste et al [42]. The rates of positivity to individual allergens with the SPT and the specific IgE tests were also consistently higher in our OFG groups. Furthermore there was a good correlation between the SPT results and the specific IgE results in individual patients, providing an objective internal control for these results.

The total serum IgE in allergic OFG patients has also been found to be elevated, significantly more so than in patients with symptoms of nasal allergy [42]. This is with or without the presence of concomitant CD. Taken together this shows that parameters of clinical allergy in OFG are elevated above levels seen in a typical allergic disorder causing nasal symptoms. Although these are observational data, they could imply an involvement of allergy in the pathogenesis of OFG or vice versa. The data also suggest there may be an overlap between OFG and OAS and that some patients with OFG may benefit from exclusion of foods which are implicated in OAS, above any current dietary therapies used in the treatment of OFG alone.

The high incidence of atopy in our cohort is not fully understood and we have no definitive evidence of SPT and food substances. Recently, a small study in which OFG patients with positive SPTs excluded cross reactive foods showed that 2/14 patients responded very well and required no further treatment [43]. Based on these findings, we do not recommend routine SPTs in OFG patients and dietary intervention cannot be recommended routinely, although a small number of patients do seem to have an excellent response.

We do acknowledge some small limitations in our study. The recent literature on cinnamon and benzoates relates to type IV hypersensitivity reactions, however, we chose to perform SPT as the logistics of performing patch testing was not possible in our clinic. Delayed late phase reactions were not sought. Oral exposure also appears to be relevant, however, SPTs were chosen to be performed as we wished to distinguish between atopic and non-atopic patients.

Future studies may be designed to address these areas.

In conclusion, we provide strong clinical evidence that patients with OFG have a strong allergic phenotype. Alongside the histological changes noted by our group, this may play a significant role in bridging the observed specific hypersensitivity to allergens and pathogenesis of OFG. The value of dietary interventions in OFG has already been demonstrated with the successful use of a cinnamon- and benzoate-free diet [17]. Our results suggest that the identification of other specific food triggers may lead to more successful individualized dietary treatments for this difficult condition. We also provide interesting data on the increased prevalence of allergy in patients with IBD without oral involvement. The significance of this is unclear and may simply reflect genetic linkages between IBD and allergy. Further studies with larger cohorts of patients are necessary to gain further insight and for validation of this association.

### Consent

Written informed consent was obtained from the patients for the publication of this report and any accompanying images.

## Competing interest

The authors declare that they have no competing interests.

## Authors’ contribution

JB initiated the project, was one of the clinical assessors and had an editorial role in writing the paper. PP carried out the studies and drafted the manuscript. All other authors were involved in sample collection and in drafting the manuscript. All authors read and approved the final manuscript.
